# Involuntary isolation: interpreting mental health legislation during the COVID-19 pandemic

**DOI:** 10.1192/bjp.2021.94

**Published:** 2021-06-23

**Authors:** Saadia Sediqzadah, Lwam Ghebrehariat, Kristen T. Weersink, David N. Fisman, Kendra A. Naidoo

**Affiliations:** 1 Department of Psychiatry, St Michael's Hospital, Toronto; Li Ka Shing Knowledge Institute of St. Michael's Hospital, Toronto; and Department of Psychiatry, University of Toronto, Canada; 2 Department of Psychiatry, University of Toronto, Canada; 3 Department of Emergency Medicine, St Michael's Hospital, Toronto; and Department of Psychiatry, University of Toronto, Canada; 4 Dalla Lana School of Public Health, University of Toronto, Canada; 5 Centre for Addiction and Mental Health, Toronto, Canada

**Keywords:** Psychiatry and law, psychiatry and public health, psychiatry and infectious disease, psychiatry and COVID19, psychiatry and health policy

## Abstract

The coronavirus disease 2019 (COVID-19) pandemic poses new and unprecedented challenges to the interpretation of mental health law. The authors present pragmatic and ethical considerations in the psychiatric safety assessment at the intersection of COVID-19 and severe mental illness.

Robert is a 37-year-old South Asian male with a history of schizoaffective disorder, bipolar type. Despite multiple medication trials, his disorder has been refractory to treatment. At his baseline, he often appears hypomanic, including loud voice, pressured speech and intrusiveness. It is not unlike him to ‘high-five’ strangers on the street. He is consistently on the verge of an involuntary psychiatric admission. You are his out-patient psychiatrist, and you and your team of allied healthcare professionals are trying your best to help him remain stable and supported in the community. He is on a community treatment order. He has developed signs and symptoms consistent with coronavirus disease 2019 (COVID-19). Despite advice from a mental health nurse, he is not self-isolating, continues to be intrusive with strangers and refuses to be tested for COVID-19. What should you do?

Different countries and jurisdictions vary widely in their approach to mental health legislation, but most share a common goal: balancing the rights and safety of people living with mental disorders while protecting public safety. Striking a balance between individual rights and public safety is also a feature of public health statutes used to control the spread of communicable diseases. The COVID-19 pandemic presents new and unprecedented challenges to the interpretation of mental health law. Assessments of safety and risk take on new meaning at the intersection of severe mental illness and infectious disease control. As per the opening fictitious vignette, what is a physician's responsibility, to the patient and the public, when an individual with a severe mental disorder is suspected to have COVID-19 and is not capable of following self-isolation practices?

Mental health legislation permits intervention when there is a risk to the safety of an individual who is or may be suffering from a mental disorder. The interpretation of ‘risk’ takes on an added layer of complexity during the COVID-19 pandemic. Certainly, the intersection between mental disorder and infectious diseases is not new, particularly as it pertains to self-care and risk of transmission (e.g. HIV, hepatitis C). However, it is the aerosol transmission, rampant community spread and (in some countries) a slow vaccination roll out that make the current situation with COVID-19 unique and presents significant challenges to individual and population health.

## Involuntary psychiatric admission

Physicians have an ethical, professional and legal responsibility to mitigate the risk of harm to the patient and the public, and an involuntary psychiatric admission may be one way of doing so. The criteria for an involuntary psychiatric admission depend on local mental health legislation; broadly, they typically include risk of harm to self or others and inability to care for self owing to mental illness. In the case of a person with a severe mental disorder who is showing signs of, or tests positive for, COVID-19 and fails to self-isolate (i.e. their mental disorder affects their insight and judgement), an involuntary psychiatric admission *may* be a unique mechanism for infectious disease control. However, is this an appropriate interpretation of mental health legislation?

As involuntary admission is an extreme measure, many jurisdictions have safeguards in place to ensure that this mechanism is used appropriately. For example, most require a probable cause to believe that a known or suspected mental disorder is giving rise to behaviours that raise risk of harm. Not all risky behaviours are a direct result of a mental disorder for people who live with these conditions. If a probable cause cannot be established, an involuntary psychiatric admission is inappropriate.

For example, UK mental health legislation (Mental Health Act 1983, section 4(2)) permits involuntary hospital admission where a person is suffering from a mental disorder and it is ‘necessary for the health or safety of the patient or protection of other persons’ that they receive treatment in a hospital. Similarly, in Ontario, Canada, an involuntary admission may be initiated if a person is suffering from a mental disorder that is likely to result in ‘serious bodily harm to others or serious physical impairment of the person’ if they are not detained in a psychiatric facility (Mental Health Act, RSO 1990, c. M. 7, s. 20(5)). A key distinction between the two jurisdictions is that, in the UK, the involuntary admission must be for the purpose of providing treatment for the mental disorder; a person cannot be involuntarily admitted solely for the purpose of treatment for a physical health condition. Further, individuals detained under the UK legislation may be treated without their consent for a certain period of time, provided that the treatment in question is not one that is specifically excluded (e.g. electroconvulsive therapy) and the treatment is given by or under the direction of an approved clinician. Applying this to Robert's case, if his mental disorder is directly resulting in his non-compliance with public health requirements and/or is jeopardising his physical health by preventing him from seeking appropriate treatment, then an involuntary admission may be considered in order to treat the mental disorder that is itself causing the COVID-19-related risk of harm to Robert or others.

On the other hand, Ontario legislation does not require that the admission be for the purpose of treating a mental disorder and an individual in Ontario cannot be treated without consent of either the individual themselves or their substitute decision maker, if the individual is deemed incapable. In Robert's case, Ontario may permit an involuntary admission if a direct link can be drawn between Robert's mental disorder and the risk to Robert's safety or the safety of others due to COVID-19. Whether Robert could be treated would depend on the issue of consent. As with any other patient, however, consideration would have to be given to weighing the various risks and benefits of an involuntary hospital admission prior to taking this significant step, as described below.

## Weighing the risks and benefits

First, let us consider the ‘risk of harm’ associated with COVID-19. According to the World Health Organization's COVID-19 Dashboard (covid19.who.int/), as of May 2021, confirmed cases exceeded 152 million globally, with over 3.2 million deaths attributed to the virus. Although these numbers are staggering, it is clear that not everyone who contracts the virus will die from it. However, mortality is only one marker of disease outcomes; we must also consider the morbidity and quality of life after infection (e.g. COVID ‘long-haulers’ or ‘long COVID’), which remain unclear. It will take years before we can appreciate the long-term health sequelae of the infection. This complicates the question of ‘risk of harm’ when considering the use of mental health legislation to involuntarily admit someone for either containment or treatment. Another risk of harm in an involuntary admission is the potential for outbreaks within the hospital if infection control practices are not followed or there is limited personal protective equipment.

What we do know is that there are certain risk factors that raise the risk of mortality. According to one study in the UK using National Health Service (NHS) data, male gender, age over 60, having diabetes, asthma or another chronic health condition, and being Black or South Asian are all important risk factors for mortality associated with COVID-19.^1^ We know that people living with severe mental illness are at greater risk of physical health conditions, including diabetes and cardiovascular disease. Additionally, a recent study found that people with schizophrenia spectrum diagnoses had almost three times the odds of dying from COVID-19, even after adjusting for comorbid medical risk factors.^2^ All of this information must be taken into consideration when assessing the risk of harm to an individual with a severe mental illness who is felt to be unable to care for themselves, with probable or known COVID-19. These risk factors will also need to be considered when assessing risk of harm to others. If Robert is not capable of self-isolating as a result of his treatment-refractory mental illness and resides in a shared living space with older people with chronic health conditions, should you use the power assigned to you as a psychiatrist to involuntarily admit Robert to mitigate the risk of harm to others?

The responsibility of the psychiatrist to mitigate risk of harm to others must be balanced with the right to autonomy of the person living with a severe mental illness. An involuntary psychiatric admission is ultimately an inherent infringement of specific rights, including the right to liberty; therefore, it should be used as a last resort. Wherever possible, a person with a severe mental disorder should be supported to live independently in the community, as is the case for Robert. Community treatment orders (CTOs) are another example of mental health legislation that is used to mitigate the risk of harm to self and others, with the difference being that this is out-patient rather than in-patient treatment. However, can a CTO enforce self-isolation or even wearing a mask? As in the case of Robert, who is consistently hypomanic and intrusive despite treatment, what can a CTO do to prevent him from high-fiving people on the street? Very little. An involuntary psychiatric admission may be the only way to minimise his COVID-19 spread in the community and to monitor his health status while infected.

## Applying the law

A physician may rely on public health statutes, or communicable disease law, for involuntary detention at either a hospital or another governmental facility, if available in their jurisdiction. The challenge with this route is that communicable disease law is often outdated and lacks clarity on how to enforce it. For example, Monaghan argues that the England and Wales Public Health (Control of Disease) Act 1984 and the Public Health (Infectious Diseases) Regulations 1988 rely on statutes dating back to 1870 and ‘many potentially useful powers are absent and the powers that exist are inflexible.’^3^ Consequently, unintuitively, mental health legislation may prove to be a more contemporary and effective way to manage infectious disease spread, where there is probable cause to believe that a person's COVID risk to themselves or others is a result of their mental illness.

Finally, an important ethical consideration for the physician is whether they are unintentionally using mental health legislation punitively to rectify social failures of society, such as poverty and homelessness. These social determinants of health are not only important precipitating and perpetuating factors for mental illness, they also raise the risk of spread of COVID-19 in these vulnerable populations. The trickle-down effects of these social failures may inadvertently force physicians to use mental health legislation to infringe on the rights of people living with mental disorders. As public health experts and the government consider how to manage spread in the homeless population (which has an overrepresentation of people with severe mental illness), they are faced with increasing pressure to improve access to adequate housing and liveable income. This is an important policy window for the medical community to advocate for the social needs of their patients.

## Conclusions

The COVID-19 pandemic presents new challenges and opportunities in interpreting mental health legislation in the context of infectious disease control. The risk assessment will be case by case and should include risk factors for mortality associated with COVID-19. To mitigate risk of harm, efforts should be made to use the least restrictive and intrusive means. Involuntary psychiatric admission for the purpose of infectious disease control may be warranted if there is a probable cause between a person's behaviour (inability to self-isolate) and their mental disorder, and other efforts to support the individual feasibly in the community have been exhausted. While the COVID-19 pandemic presents challenges, it also presents opportunities for creative solutions and advocacy efforts for the social determinants of health for patients.
